# Determinants of suboptimal breastfeeding practices in Nigeria: evidence from the 2008 demographic and health survey

**DOI:** 10.1186/s12889-015-1595-7

**Published:** 2015-03-18

**Authors:** Felix A Ogbo, Kingsley E Agho, Andrew Page

**Affiliations:** School of Science and Health, University of Western Sydney, Campbelltown Campus, Locked Bag 1797, Penrith, NSW 2571 Australia

**Keywords:** Breastfeeding, Determinants, Mortality, Nigeria, Suboptimal

## Abstract

**Background:**

In Nigeria, suboptimal breastfeeding practices are contributing to the burden of childhood diseases and mortality. This study identified the determinants of key suboptimal breastfeeding practices among children 0–23 months in Nigeria.

**Method:**

Data on 10,225 children under-24 months were obtained from the 2008 Nigeria Demographic and Health Survey (NDHS). Socio-economic, health service and individual factors associated with key breastfeeding indicators (early initiation of breastfeeding, exclusive breastfeeding, predominant breastfeeding and bottle feeding) were investigated using multiple logistic regression analyses.

**Results:**

Among infants 0–5 months of age, 14% [95% confidence Interval (CI): 13%, 15%] were exclusively breastfed and 48% [95% CI: 46, 50%] were predominantly breastfed. Among children aged 0–23 months, 38% [95% CI 36, 39%] were breastfed within the first hour of birth, and 15% [95% CI: 14, 17%] were bottle-fed. Early initiation of breastfeeding was associated with higher maternal education, frequent antenatal care (ANC) visits and birth interval but deliveries at a health facility with caesarean section was associated with delayed initiation of breastfeeding. Educated mothers, older mothers and mothers from wealthier households exclusively breastfeed their babies. The risk for bottle feeding was higher among educated mothers and fathers, and women from wealthier households including mothers who made frequent ANC visits.

**Conclusion:**

Socio-economic and health service factors were associated with suboptimal breastfeeding practices in Nigeria. To improve the current breastfeeding practices, breastfeeding initiatives should target all mothers – particularly low SES mothers – including, national and sub-national health policies that ensure improved access to maternal health services, and improvements to baby friendly hospital and community initiatives for mothers.

**Electronic supplementary material:**

The online version of this article (doi:10.1186/s12889-015-1595-7) contains supplementary material, which is available to authorized users.

## Background

Optimal breastfeeding practices play a key role in improving the health and development of children under-5 years, and have been shown to be associated with decreased risk of childhood diarrhoea, and respiratory tract infections as well as reductions in childhood mortality [1-5]. Current recommendations for breastfeeding practices include the initiation of breastfeeding for all newborns within the first hour of life, exclusive breastfeeding (EBF) for the first 6 months of life, and continued breastfeeding for two years and beyond with nutritionally appropriate and safe complementary foods introduced at the sixth month [6-9].

Globally, only 38% of infants are exclusively breastfed for the first four months of life [3,10], and recent analyses found that over 800, 000 deaths [11] and about 10% of the global burden of disease among children under-5 years in developing countries resulted from suboptimal breastfeeding practices [12]. In 1992, following global recommendations, Nigeria introduced the baby friendly hospital initiative (BFHI) [13] which resulted in some improvement in early initiation of breastfeeding (33%) [14], however, the duration and practice of exclusive breastfeeding remains low (17%) and falls short of the estimated levels required to achieve substantial declines in childhood mortality [15-17]. In addition, the prevalence of continued breastfeeding at one year in Nigeria is high (84%) [14] compared to the United States (26%) [18] but continued breastfeeding at two years is low (35%) in Nigeria [14]. Among sub-Saharan Africa countries, the prevalence of exclusive breastfeeding in Nigeria remains one of the lowest (17%) compared to other nations like Tanzania (50%) [19] and Kenya (32%) [20]. Breastfeeding practices are crucial to the achievement of millennium development goal four (MDG-4), but available evidence shows that the goal has not been achieved in Nigeria [21].

In Nigeria, several studies have identified various factors limiting optimal breastfeeding practices [15,16,22-24]. These studies were conducted in various regions of Nigeria, and focused on EBF and delayed initiation of breastfeeding [17,22,24-27]. Findings from these studies found that individual factors (such as sex of the baby, work, the mother’s age and family pressures) [16,26-28], health service factors (such as antenatal visits, delivery at the government hospital and mode of delivery) [17,23], and household wealth and geopolitical differences [16] were associated with non-EBF and delayed initiation of breastfeeding.

To date, there has been no nationally representative study of suboptimal breastfeeding practices, that incorporate key outcome measures such as predominant breastfeeding and bottle feeding [2,3,5,6] nor has there been an inclusive assessment of the determinants of suboptimal breastfeeding patterns at the national level in Nigeria. Accordingly, the main purpose of this study is to identify socio-economic, health service and individual determinants of key optimal breastfeeding practices in Nigeria using the 2008 Demographic and Health Survey for Nigeria. Results from this study will provide an evidence-based assessment of infant and young child feeding practices to inform policy makers and public health workers to frame policies and programs that would improve breastfeeding practices in Nigeria.

## Methods

### Data source

The analysis was based on the publicly available dataset collected for the Nigeria Demographic and Health Survey (NDHS) 2008, conducted by the National Population Commission (NPC) and ICF International [29]. The NDHS collects information on optimal breastfeeding (among other factors) from a nationally representative sample of households. The sample was selected in a stratified two-stage cluster design. A total of 34,070 eligible women – all women aged 15–49 years who were either permanent residents of the households or visitors present in the households on the night before the survey – were interviewed (response rate 98%), using a questionnaire to collect data on respondent’s demographics, maternal and child health practices as well as reproductive, contraceptive and infant feeding practices [29]. Our analysis was restricted to the youngest living children aged less than 24 months, living with respondent (eligible women aged 15–49 years), and the total weighted sample was 10,225.

#### Key breastfeeding indicators

The infant feeding indicators were assessed using the World Health Organization recommended definition of breastfeeding indicators for assessing Infant and Young Child Feeding (IYCF) practices [3,30,31]. In the analysis, the main outcome factors were (i) early initiation of breastfeeding, (ii) exclusive breastfeeding up to 6 months, (iii) predominant breastfeeding and (iv) bottle feeding, using the following definitions:

- Early initiation of breastfeeding: The proportion of children 0–23 months of age who were put to the breast within one hour of birth – this indicator was based on historical recall.
- Exclusive breastfeeding: The proportion of infants 0–5 months of age who received breast milk as the only source of nourishment (but which allows oral rehydration solution, drops or syrups of vitamins and medicines) – this indicator was based on mother’s recall on feeds given to the infant in the last 24 hours.
- Predominant breastfeeding: The proportion of infants 0–5 months of age who received breast milk as the predominant source of nourishment (but which allows water and water-based drinks fruit juice, ritual fluids, oral rehydration solution, syrups or drops of vitamins) during the previous day.
- Bottle-feeding: The proportion of infants 0–23 months of age who received any liquid (including breast milk) or semi-solid food from a bottle with nipple/teat.

Exclusive breastfeeding (EBF) and early initiation of breastfeeding were included in the analyses because of their association with improved infant nutrition, decreased morbidity and mortality among children under-5 years [1,11,12]. Predominant breastfeeding and bottle-feeding were included due to their impacts on the increased risk of diarrhoeal and respiratory illness including increased risk for infant mortality [2,3,5,6]. Continued breastfeeding at one and two years were not examined in this analysis, and suboptimal breastfeeding practices were defined as feeding behaviours that did not meet recommended standards.

#### Study factors

The study factors included socio-economic, health service and individual factors. Socio-economic characteristics included the mother’s highest educational level (categorized as no education, primary or secondary and above education) and employment status (categorized as not working or working), household wealth index (categorized as poor, middle or rich), father’s highest educational level (categorized as no education, primary or secondary and above education) and geographical region. The household wealth index was calculated as a score of household assets which was derived from a principal components analysis conducted by the National Population Commission (NPC) and ICF International based on a methodology developed from previous DHSs [32,33], and using methods recommended by the World Bank Poverty Network and UNICEF [34]. The geographical regions were grouped into six geographical zones: North-east, North-west, North-central, South-east, South-west and South-south. The zones are constitutionally recognised regions of Nigeria based on geographical locations of various states and the federal capital territory [29].

Health service factors included the number of antenatal clinic visits (categorized as no antenatal visit, one to three antenatal visit or four and above antenatal visits), the place of delivery (home or health facility) and mode of delivery (caesarean section or vaginal) the type of delivery assistance, the timing of postnatal visits (categorized as no post natal visits, 0–2 days, 3–6 days or above 7 days postnatal visits). Type of delivery assistance received was categorized as either a health professional or non-health professional e.g. a traditional birth attendant. A traditional birth attendant is usually a woman, who assists the mother during childbirth and who initially acquired her skills by delivering babies herself or by working with other traditional birth attendants [35].

Individual characteristics included maternal age (categorized as 15–24 years, 25–34 years or 35–49 years), age of the child, sex of the child (categorized as male or female), birth order of child (the position of the under-5 child in the family) and preceding birth interval (categorized as no previous births, less 24 months or more than or equal to 24 months birth interval).

Other study factors considered in the analysis included marital status, perceived size of baby, maternal body mass index, place of residence, frequency of listening to radio, frequency of watching television and frequency of reading newspaper. However, these factors were excluded following preliminary analyses as no significant associations between breastfeeding indicators and the study factors were apparent in univariate analyses.

### Statistical analysis

The selected breastfeeding feeding indicators (early initiation of breastfeeding, exclusive breastfeeding, predominant breastfeeding and bottle-feeding) were expressed as dichotomous outcomes. Prevalences were calculated using Stata (Stata Corp 12.0) with the ‘Svy’ function used to allow for adjustments for the cluster sampling design, sampling weights and the calculation of standard errors. The Taylor series linearization method was used in the surveys when estimating confidence intervals around prevalence estimates. Univariable and multivariable logistic regression methods were used to investigate relative associations between socio-economic factors, health service and individual factors, and breastfeeding indicators.

A stepwise backward elimination approach was applied, which was validated *post hoc* by using the following procedures: (1) we initially entered main risk factors (socio-economic, health service and individual) [16,17,19,22,23] with P-value <0.20 in the backward elimination process, (2) we tested the backward elimination by also including all variables (all potential risk factors); and, (3) we tested and reported any collinearity between study factors in the final model. Odds ratios and 95% CIs were calculated to assess the association between study factors and breastfeeding indicators.

Multivariable models adjusted for the potential confounding factors of marital status, perceived size of baby, maternal body mass index, place of residence, frequency of listening to radio, frequency of watching television and frequency of reading newspaper [36]. In models of health service factors, additional adjustment was made for socio-economic and individual factors, as a confounder of the association between health service factors and breastfeeding indicators. Similarly, for models of individual characteristics, additional adjustment was made for socio-economic and health service factors. Also, we combined the place and mode of delivery to see the effect of caesarean deliveries and home deliveries on early initiation of breastfeeding [17,19,37,38].

### Ethics

The DHS project sought and obtained the required ethical approval from the National Health Research Ethic Committee (NHREC) in Nigeria before the surveys were conducted. Informed consent was obtained from study participants before they were allowed to participate in the surveys. The survey data sets used in this study was based on publicly available dataset that is freely available online with no participant’s identity. Approval was sought from MEASURE DHS/ ICF International and permission was granted for this use.

## Results

### Characteristics of the study population

Most of the children lived in rural areas (70%). Approximately 33% of the mothers finished secondary or higher level of education, and 61% were employed in the last 12 months. Approximately 62% of the total births took place at home and the proportion of caesarean deliveries was low in comparison (1.7%) [Additional file 1: Table S1]. Female and male children were almost equally distributed in the sample, and approximately 55% of pregnant mothers made more than one antenatal visit, and only 42% of mothers had a postnatal check-up after 6 weeks. The majority of the children were from the North-west (31%), South-south (17%) and North-east (16%) regions of the country.

### Exclusive breastfeeding

The prevalence of exclusive breastfeeding for infants was 13.5% [95% CI: 12, 15%] (Table 1). Mothers from wealthier households were more likely to exclusively breastfeed their babies compared to mothers from poorer households (OR = 1.62, 95%CI: 1.14, 2.31; p = 0.008 for middle income households; OR = 1.64, 95%CI: 1.04; 2.60 p = 0.034 for high income households) [Table 2]. In the final model, household wealth was replaced with mother’s education which showed that educated mothers were more likely to exclusively breastfeed their babies compared to mothers with no schooling (OR = 1.87, 95%CI: 1.24, 2.82; P = 0.003 for secondary and above). Similarly, babies whose mother’s age was between 25–34 years were more likely to be exclusively breastfeed compared to mothers in the youngest age group (OR = 1.50; 95%CI: 1.12; 2.01; P = 0.007). The odds for exclusive breastfeeding were lower among babies in the north-east (OR = 0.23, 95%CI: 0.12, 0.34; P < 0.001), north-west (OR = 0.43, 95%CI: 0.25, 0.74, p = 0.002), south-east (OR = 0.41, 95%CI: 0.26, 0.65; P < 0.001), and south-west (OR = 0.54, 95%CI: 0.35, 0.83; p = 0.005) compared to children in the north-central region.

**Table 1:** **Key breastfeeding indicators among children 0–23 months, Nigeria 2008 (n = 10225)**

**Indicators**	Sample size N* (weighted)	**n† (unweighted)**	**Rate (%)**	**95% CI**	
Early initiation of breastfeeding rate (0–23 months)(0–11 months)	5604	2116	37.80	35.90	39.70
(12–23 months)	4621	1738	37.62	35.52	39.76
(0–23 months)	10225	3854	37.70	36.01	39.41
Exclusive breastfeeding rate (0–5 months)(0–1 months)	738	149	20.18	17.08	23.67
(2–3 months)	1011	146	14.42	12.11	17.07
(4–5 months)	1083	86	7.96	6.28	10.04
(0–3 months)	1749	295	16.85	14.78	19.14
(0–5 months)	2832	381	13.45	11.89	15.17
Predominant breastfeeding rate (0–5 months)	2832	1361	48.08	45.70	50.47
Bottle-feeding rate (0–5 months)					
(0–5 months)	2832	433	15.29	13.68	17.05
(6–11 months)	2772	372	13.40	11.96	14.99
(12–23 months)	4621	338	7.32	6.46	8.29
(0–23 months)	10225	1143	11.18	10.34	12.07

N* = Population size n† = Positive cases.

***(i) Early initiation of breastfeeding:*** The proportion of children 0–23 months of age who were put to the breast within one hour of birth.

***(i) Exclusive Breastfeeding:*** The proportion of infants 0–5 months of age who received breast milk as the only source of nourishment (but allows oral rehydration solution, drops or syrups of vitamins and medicines).

***(iii) Predominant breastfeeding:*** The proportion of infants 0–5 months of age who received breast milk as the predominant source of nourishment (but which allows water and water-based drinks fruit juice, ritual fluids, oral rehydration solution, syrups or drops of vitamins).

***(iv) Bottle-feeding:*** The proportion of infants 0–23 months of age who received any liquid (including breast milk) or semi-solid food from a bottle with nipple/teat.

**Table 2:** **Determinants of Exclusive breastfeeding and predominant breastfeeding in Nigerian women (2008)**

		**Exclusively breastfeeding**					**Predominant breastfeeding**				
	**Sample**	**(%)**	**Unadjusted**		**Adjusted**		**%**	**Unadjusted**		**Adjusted**	
	size (n*)		**OR (LCI-UCI)**	**P**	**OR (LCI-UCI)**	**P**		**OR (LCI-UCI)**	**P**	**OR (LCI-UCI)**	**P**
*Socio-economic*											
**Maternal education**											
No education	1249	7.4	1.00		1.00		55.9	1.00		1.00	
Primary	625	14.0	2.03 (1.42-2.91)	<0.001	1.34 (0.90-2.00)	0.148	45.1	0.65 (0.52-0.81)	0.010	0.7 (0.55-0.89)	0.004
Secondary and above	958	21.0	3.32 (2.34-4.72)	<0.001	1.87 (1.24-2.82)	0.003	39.8	0.52 (0.42-0.65)	0.010	0.57 (0.45-0.72)	<0.001
**Mother’s age**											
15-24 years	947	10.3	1.00		1.00		50.6	1.00			
25-34 years	1360	16.3	1.68 (1.28-2.22)	<0.001	1.50 (1.12-2.01)	0.007	46.9	0.86 (0.71-1.04)	0.123		
35-49 years	524	11.7	1.15 (0.80-1.64)	0.439	1.17 (0.89-1.70)	0.379	46.6	0.85 (0.68-1.07)	0.167		
**Household Wealth**											
Poor	1295	15.5	1.00		1.00		86.7	1.00			
Middle	1046	33.3	2.39 (1.73-3.30)	<0.001	1.62 (1.14-2.31)	0.008	87.6	0.67 (0.55-0.81)	0.001		
Rich	491	21.9	3.36 (2.31-4.88)	<0.001	1.64 (1.04-2.60)	0.034	41.6	0.61 (0.46-0.8)	0.001		
**Geopolitical region**											
North Central	380	21.6	1.00		1.00		30.6	1.00			
North East	496	4.0	0.15 (0.09-0.25)	<0.001	0.23 (0.12-0.34)	<0.001	60.5	3.47 (2.5-4.81)	0.010		
North West	817	8.4	0.33(0.20-0.56)	<0.001	0.43 (0.25-0.74)	0.002	62.8	3.82 (2.87-5.09)	0.010		
South East	271	14.9	0.63 (0.41-0.99)	0.043	0.41 (0.26-0.65)	<0.001	42.3	1.66 (1.19-2.32)	0.003		
South West	393	16.5	0.72 (0.47-1.10)	0.129	0.54 (0.35-0.83)	0.005	28.2	0.89 (0.64-1.25)	0.502		
South South	475	22.3	1.04 (0.70-1.56)	0.840	0.73 (0.46-1.13)	0.157	43.5	1.74 (1.27-2.4)	0.001		
*Individual*											
**Child age in months**	2832	13.5	1.3 (1.21-1.4)	0.001	0.75 (0.70-0.82)	<0.001	48.1	0.85 (0.81-0.9)	0.001	0.85 (0.8-0.89)	<0.001
*Health service*											
**Antenatal visits**											
None	1250	9.1	1.00				54.7	1.00		1.00	
1-3	344	11.2	1.26 (0.80-1.99)	0.317			43.1	0.63 (0.48-0.82)	0.001	0.67 (0.51-0.88)	0.004
≥4	1238	18.4	2.25 (1.66-3.05)	<0.001			42.8	0.62 (0.51-0.75)	0.001	0.78 (0.63-0.97)	0.027

n* = Children’s age 0–5 months.

*Statistically significant (P < 0.05) study factors from multivariate models are shown. Adjustments were done for marital status, perceived size of baby, maternal body mass index, place of residence, frequency of listening to radio, frequency of watching television and frequency of reading newspaper. In models of socio-economic factors, adjustments were made for health service and individual factors. In models of health service factors, additional adjustment was made for socio-economic status and further control of socio-economic and health service factors was done for Individual factors.*

### Predominant breastfeeding

Almost half [48%, 95% CI: 46, 50%] of children aged 0–5 months were predominantly breastfed [Table 1]. The odds for predominant breastfeeding were significantly lower among mothers who had 1–3 antenatal visits (OR = 0.68, 95% CI: 0.52, 0.89; p = 0.004), and those who had four or more visits (OR = 0.78, 95% CI: 0.63, 0.97; P = 0.027) compared to mothers who had no antenatal visit [Table 2]. Mothers who had primary (OR = 0.70, 95%CI: 0.55, 0.89; P = 0.004), secondary or higher education (OR = 0.57, 95% CI: 0.45, 0.72; P < 0.001) were significantly less likely to predominantly breastfeed their babies compared to mothers with no education.

### Early initiation of breastfeeding

Less than half of the mothers [38.0%, 95%CI: 36, 39%] initiated breastfeeding, within the first hour of birth [Table 1]. Educated mothers were significantly more likely to initiate breastfeeding early compared to mothers with no education (OR = 1.43, 95%CI: 1.21, 1.65; p < 0.001 for primary education and OR = 1.46, 95%CI: 1.24, 1.72; p < 0.001 for secondary and above) [Table 3]. Similarly, mothers who delivered their babies at a health facility by caesarean section (OR = 0.48, 95%CI: 0.31, 0.75; p = 0.001) were significantly less likely to initiate breastfeeding compared to mothers who delivered their babies vaginally. Babies of educated fathers were more likely to be breastfed, within the first hour of birth compared to babies whose fathers had no education [Table 3]. Higher birth order was also associated with early initiation of breastfeeding. In the final model, birth order was replaced with preceding birth interval, and the analysis showed that mothers whose preceding birth interval was equal to or greater than 24 months were significantly more likely to initiate breastfeeding compared to mothers with no previous birth (OR = 1.25, 95% CI: 1.09, 1.44; p < 0.001).

**Table 3:** **Determinants of early initiation of breastfeeding and bottle feeding in Nigerian women (2008)**

		**Early initiation of breastfeeding**					**Bottle feeding**				
	**Sample**	**(%)**	**Unadjusted**		**Adjusted**		**%**	**Unadjusted**		**Adjusted**	
	Size (n*)		**OR (LCI-UCI)**	**P**	**OR (LCI-UCI)**	**P**		**OR (LCI-UCI)**	**P**	**OR (LCI-UCI)**	**P**
*Socio-economic*											
**Maternal education**											
No education	4655	30.4	1.00		1.00		4.4	1.00		1.00	
Primary	2274	43.7	1.77 (1.52-2.07)	<0.001	1.43 (1.21-1.65)	<0.001	13.4	3.34 (2.71-4.12)	0.001	1.44 (1.12-1.84)	0.005
Secondary and above	3296	43.9	1.79 (1.55-2.07)	<0.001	1.46 (1.24-1.72)	<0.001	19.2	5.15 (4.24-6.26)	0.001	1.41 (1.06-1.88)	0.017
**Father’s education**											
No education	3641	30.3	1.00				3.7	1.00		1.00	
Primary	2149	39.1	1.48 (1.26-1.74)	<0.001			12.5	3.71 (2.88-4.77)	0.001	1.81 (1.37-2.38)	<0.001
Secondary and above	4044	43.8	1.80 (1.55-2.08)	<0.001			17.0	5.31 (4.25-6.63)	0.001	1.84 (1.39-2.43)	<0.001
**Household Wealth**											
Poor	4709	32.8	1.00				5.9	1.00		1.00	
Middle	3749	41.8	1.47(1.28-1.68)	<0.001			13.3	2.43 (2.01-2.94)	0.001	1.23 (0.99-1.52)	0.062
Rich	1767	42.1	1.49 (1.25-1.79)	<0.001			20.5	4.09 (3.28-5.10)	0.001	1.45 (1.09-1.92)	0.010
**Geopolitical region**											
North Central	3112	59.6	1.00		1.00		11.1	1.00		1.00	
North East	5019	23.8	0.21 (0.16-0.28)	<0.001	0.24 (3.12-5.45)	<0.001	6.8	0.59 (0.42-0.81)	0.001	0.93 (0.67-1.29)	0.660
North West	2093	30.6	0.30 (0.24-0.38)	<0.001	0.35 (2.22-3.59)	<0.001	4.1	0.34 (0.24-0.48)	0.001	0.56 (0.40-0.79)	0.001
South East	3112	38.3	0.42 (0.33-0.54)	<0.001	0.34 (2.22-3.70)	<0.001	20.6	2.07 (1.53-2.81)	0.001	1.50 (1.10-2.05)	0.010
South West	5019	50.2	0.68 (0.52-0.90)	0.007	0.35 (1.25-2.16)	<0.001	19.5	1.94 (1.44-2.62)	0.001	1.45 (1.06-1.98)	0.020
South South	2093	36.7	0.39 (0.30-0.50)	<0.001	0.61 (2.31-3.75)	<0.001	16.5	1.58 (1.18-2.13)	0.002	1.03 (0.76-1.40)	0.832
*Individual*											
**Child age in months**											
**Age of child (months)**	10225	37.7	1.00 (0.10-1.00)	0.483			10.7	0.94 (0.93-0.96)	0.001	0.94 (0.93-0.95)	<0.001
**Birth order**											
First-born	1953	34.4	1.00				15.8	1.00			
2nd-4^th^	4703	38.7	1.20 (1.06-1.36)	0.003	1.26 (1.11-1.43)	<0.001	11.9	0.72 (0.60-0.86)	0.001		
5 or more	3569	38.1	1.17 (1.03-1.33)	0.016	1.41 (1.23-1.62)	<0.001	7.7	0.44 (0.37-0.54)	0.001		
**Preceding birth interval**											
No previous birth	1953	34.4	1.00		1.00		15.8	1.00		1.00	
<24 months	1591	36.5	1.09 (0.93-1.28)	0.261	1.13 (0.95.1.34)	0.159	11.7	0.71 (0.56-0.90)	0.004	0.79 (0.62-1.02)	0.074
≥24 months	6668	38.9	1.21 (1.08-1.36)	0.001	1.25 (1.09-1.44)	0.001	9.7	0.57 (0.48-0.67)	0.001	0.68 (0.57-0.82)	<0.001
*Health service*											
**Combined place and mode of delivery**											
Home	6563	37.7	1.00		1.00		13.1	1.00		1.00	
Health facility, non-caesarean	3481	37.9	1.60 (1.41-1.82)	<0.001	1.26 (1.09-1.44)	0.001	10.9	3.02 (1.63-5.58)	<0.001	1.10 (0.91-1.32)	0.322
Health facility, caesarean	175	25.5	0.66 (0.43-1.02)	0.063	0.48 (0.31-0.75)	0.001	28.8	8.78 (4.53-17.01)	<0.001	1.88 (1.23-2.89)	0.004
**Antenatal Clinic visits**											
None	4594	35.1	1.00				6.0	1.00		1.00	
1-3	1084	39.6	1.21 (1.01-1.45)	0.037			11.9	2.12 (1.66-2.71)	0.001	1.37 (1.05-1.77)	0.018
≥4	4547	39.9	1.23 (1.08-1.40)	0.002			16.3	3.05 (2.54-3.68)	0.001	1.49 (1.22-1.82)	<0.001

n* = Children’s age 0–23 months.

*Statistically significant (P < 0.05) study factors from multivariate models are shown. Adjustments were done for marital status, perceived size of baby, maternal body mass index, place of residence, frequency of listening to radio, frequency of watching television and frequency of reading newspaper. In models of socio-economic factors, adjustments were made for health service and individual factors. In models of health service factors, additional adjustment was made for socio-economic status, and further control of socio-economic and health service factors was done for Individual factors.*

### Bottle feeding

Approximately 15% [95% CI: 14, 17%] of babies were bottle-fed [Table 1], and the odds for bottle feeding were significantly higher in mothers who had primary (OR = 1.44, 95% CI: 1.12, 1.84; p < 0.005) and secondary or higher education (OR = 1.41, 95% CI: 1.06, 1.88; p < 0.017) compared to mothers with no schooling [Table 3]. Mothers who delivered at a health facility by caesarean section were significantly more likely to engage in bottle feeding compared to mothers who delivered at home (OR = 1.88, 95% CI: 1.23, 2.89; p = 0.004). Bottle feeding was higher in mothers from wealthier households compared to mothers from poorer households (OR = 1.45, 95% CI: 1.09, 1.92; p < 0.001). Higher paternal education was associated with bottle feeding compared to babies whose fathers had no schooling [Table 3]. Additionally, babies of mothers who had four or more antenatal visits were significantly more likely to be bottle-fed compared to babies delivered by mothers who had no antenatal visit (OR = 1.45, 95% CI: 1.22, 1.82; p < 0.001). Mothers who lived in the north-west (OR = 2.83, 95% CI: 2.22, 3.59; p < 0.001), south-east (OR = 2.86, 95% CI: 2.22, 3.70; p < 0.001), south-west (OR = 1.64, 95%CI: 1.25, 2.16; p < 0.001) were significantly more likely to bottle-feed their babies compared to children in the north-central region. The prevalence of bottle feeding was higher in children aged 0–12 months compared to children 13–23 months. However, sub-group analysis of children aged 0–5, 6–11, 12–17 and 18–23 months showed no association for bottle feeding.

## Discussion

Optimal breastfeeding rates (early initiation of breastfeeding and exclusive breastfeeding) were very low (38% and 13% respectively), indicating that most children under-6 months were given other foods or liquids in addition to breast milk. The prevalence of bottle-feeding and predominant breastfeeding were high (15.3% and 48.1% respectively) in children aged 0–23 months and 0–5 months, respectively – a period of development when optimal breastfeeding is most important for child health and development [2,39]. Additionally, this study found that lower maternal age and education, lower household wealth, lower frequency of antenatal visits, and caesarean delivery at a health facility were associated with feeding behaviours that did not meet recommended standards. There was also substantial geopolitical variability in breastfeeding patterns, with relatively lower prevalence in the less urbanised jurisdictions of Nigeria (North East and North West) compared to other regions.

There are a number of methodological limitations that need to be considered in interpreting findings from the current study. Firstly, breastfeeding indicators were based on self-report. This is a potential source of measurement bias in the outcome, where women may incorrectly recall how the child was fed during the periods referred to by the survey questions. Similarly, misclassification in selected study variables may also be present, for example over- or under-estimation of the number of health service visits. Selection bias is less likely to affect observed findings, due to the nationally representative sampling frame of the survey and high response rate. Selected samples were drawn from the 2006 national census frame yielding 98% response rate without significant differences between urban and rural areas. Finally, the findings are based on cross-sectional data, so it is difficult to ascribe temporality between putative exposures and outcomes. However, implied temporal associations are more logically sustainable for exposures that are categorical and fixed, and established early in life around seminal events, such as educational achievement, and some reproductive factors such as number of children and the date of birth of a child.

The findings showed that mid-reproductive age of the mother (25–34 years) was associated with exclusive breastfeeding compared to younger mothers, signifying that younger mothers may be inexperienced in conventional infant feeding practices and may be more likely to engage in suboptimal feeding practices. Similar studies in Nigeria and Canada reported lower maternal age as a significant factor for non-EBF [17,25,26,40]. A recent study in Nigeria found that counseling at the health facility was an important strategy for promoting exclusive breastfeeding practice in women [26]. Similarly, findings from an international literature review found that using breastfeeding peer support strategy was effective in ensuring exclusive breastfeeding among mothers [41]. Accordingly, these approaches could be employed in Nigeria to improve the uptake of exclusive breastfeeding among younger mothers in communities.

High maternal education was associated with a greater likelihood of exclusive breastfeeding compared to mothers with no education. The association with education became stronger after controlling for household wealth suggesting that it was not a wealth effect. Nonetheless, previous studies from developing countries reported that women of higher socio-economic status (SES) groups may be more likely to have better access and respond to health promotion messages (including infant feeding messages) compared to women of lower SES groups [42-44]. This finding is consistent with previous studies in Nigeria and Tanzania that reported low maternal education as a major determinant for non-exclusive breastfeeding [15,17,19,23,24]. Similarly, the study also found an association between mothers who had at least a primary level of education and predominant breastfeeding compared to mothers with no schooling, indicating the crucial role of mother’s education on infant nutrition and development. Studies have shown that primary education is the basic threshold required to benefit from health information, and it provides marginalised groups – particularly women – the self-confidence desired to act on health information [45]. In Nigeria, previous studies found that women with no schooling had limited knowledge and attitude towards optimal breastfeeding practices [24,27]. Therefore, continuous implementation and sustainability of the MDG project in this context is crucial to improving breastfeeding practices of Nigerian women.

Previous Nigerian studies reported various reasons for why women of low SES groups do not exclusively breastfeed their babies including, that EBF is demanding [46], a perceived notion that the child continued to be hungry after breastfeeding [27,47], lack of family support [27,46]; and women in private or public employment engaged in non-EBF due to existence of workplace barriers that do not support appropriate breastfeeding practices [48,49]. A recent study in regional Nigeria found that mothers who had contacts with a health facility received information about optimal breastfeeding practices [25]. Thus, national health policies and programs that encourage mothers of low SES groups to access health facility are recommended to improve breastfeeding patterns in Nigerian women.

Studies from Ghana and Nigeria reported that mothers who used conventional health services engaged in better breastfeeding practices [13,50]. However, in the present study, mothers who made frequent antenatal visits (ANC), predominantly breastfed and bottle-fed their babies compared to mothers who made no ANC visits. Access to a health facility provides an opportunity to obtain and respond to health promotion messages, and ANC visit present an important opportunity for implementation of appropriate infant feeding intervention strategies to promote optimal breastfeeding behaviours. Findings from this study, however, suggest that relevant messages about breastfeeding may not have been communicated effectively to mothers by antenatal staff. Similarly, mothers may have received the right messages, but socio-cultural beliefs such as a perceived notion that water is needed by the baby after breastfeeding, lack of family support [27] or pressure to resume work [28,48] influenced their decision to predominantly breastfeed or bottle-feed. For example, in many Nigerian communities, new mothers do not have the autonomy to exclusively make household decisions regarding infant and young child feeding. These decisions are often made by the father or by the grandmother (paternal or maternal) [51] acknowledging that Nigerian grandmothers usually provide significant support to nursing mothers [27], and most grandmothers are knowledgeable in infant feeding practices but these skills are often based on traditional belief systems [52]. Based on their role, they can play a large part in influencing mother’s decision to predominantly breastfeed or bottle-feed [53], and this may be an additional reason for why mothers in Nigeria, engage in suboptimal feeding practices.

Although, the association between employment status and key breastfeeding indicators was not apparent in this study, recent studies in Nigeria found that employed mothers predominantly breastfeed and bottle-feed their babies due to pressure to resume work [15,28,48], and thus, dedicate less time to optimal breastfeeding practices. Employed mothers in private or public organisations – more likely to be educated and work under rigid time schedules – may not have flexible time to engage in optimal breastfeeding practices compared to unemployed or self-employed mothers, and it appears that organisational employment may repudiate the benefits of higher education on optimal breastfeeding patterns. A response to this impediment in Nigeria has been a regional government initiative, which recently announced 10-days paid parental leave for male public servants and extended paid maternity leave for female public officers from three to six months, with the promotion of exclusive breastfeeding being part of the rationale for this intervention [54]. National policies that support nursing mothers in work environments such as provision of crèches, paid parental leave and extended paid maternal leave including strengthening of the International code of Marketing of Breast-milk Substitutes in Nigeria are proposed to improve breastfeeding outcomes.

Early initiation of breastfeeding is essential to the health and development of the infant [2,3] and its delay may be a significant risk factor for infant mortality [39]. In the present study, a birth interval of more than 24 months was associated with early initiation of breastfeeding, suggesting that family planning can promote breastfeeding practices. In Nigeria, mothers with no schooling are more likely to deliver at home compared to educated mothers, and home delivery remains an impediment to early initiation of breastfeeding [29]. Mothers with at least a primary education engaged in early initiation of breastfeeding compared to mothers with no schooling, suggesting that educated mothers were more likely to have their babies at a health facility that would require delivery supervision by a health professional [29]. Studies from Nigeria and Australia found that father’s education played an important role in optimum breastfeeding [55,56], and in the present study, babies of educated fathers were breastfed, within the first hour of birth. However, mothers who delivered their babies at a health facility by caesarean section, delayed initiation of breastfeeding compared to mothers who delivered vaginally at home without skilled personnel supervision. In the study, frequent ANC visit was associated with early initiation of breastfeeding. Similar studies done in Nigeria, Tanzania, Guatemala, Ghana and Pakistan identified mother’s education, ANC visits, mode and place of delivery as significant determinants for early initiation of breastfeeding [17,19,28,37,38,57].

Consistent with this finding, some studies done in Ghana and Nigeria [28,38], and a report from an international review [58] suggested that caesarean section and peri-natal care remained important impediments to early initiation of breastfeeding, where babies were usually handed over to the paediatrician or the paediatric nurse for care while the mother was still in post-operative or peri-natal care [28,38]. Further training of health professionals is required to ensure the wider benefits of focused antenatal care (ANC) (a four-visit ANC model) and baby friendly hospital initiatives (BFHI). However, in many developing countries like Nigeria, most deliveries occur at home, and those that deliver in the hospital return to their homes and communities for postnatal care of their babies [59]. Thus, baby friendly community initiatives (BFCI), which are community-based approaches to protect, promote and support optimal breastfeeding practices are also proposed as an adjunct to health service based initiatives [59].

Mothers from wealthier households with higher educational achievement were more likely to engage in bottle-feeding practices compared to mothers from poorer households with lower educational achievement respectively, suggesting that higher socio-economic status women – more likely to be employed in formal environments [60-62] – have the material resources to purchase formula foods. Similarly, babies of educated fathers received foods mainly from a bottle compared to babies whose fathers had no schooling, indicating that socio-cultural perceptions about breastfeeding have also engendered a preference for bottle feeding [49]. These findings were similar to those previously documented in Guatemala and India, where an association between maternal education, household wealth and bottle feeding was also reported [57,63].

Nigerian data from the Millennium Development Goals (MDGs) performance tracking survey showed that the literacy rate among women from Southern Nigeria (89% - 92%) was higher compared to women from Northern Nigeria (30% - 66%), and women from the Southern zones were more likely to transit to higher educational levels [64]. Similarly, women from Southern geopolitical regions – more educated and wealthier zones [65] – of Nigeria had better access to health care services compared to women from Northern Nigeria, and are more likely to be employed [64]. These observed differences may play an important role in the geopolitical variability in infant feeding practices identified in the current study.

## Conclusion

This study found that Nigeria’s suboptimal breastfeeding practices were associated with mothers who had no schooling, lower household income, and women who had no contacts with health services, particularly ANC visits. However, there was also a sub-group of women with greater material resources and higher education with higher levels of health service use, and a higher likelihood of birth by caesarean section who also reported suboptimal breastfeeding patterns.

Appropriate breastfeeding intervention programs focused on all mothers – especially mothers with no schooling and women from poorer households – that consider geopolitical variability could be implemented to encourage mothers to use health care services. In addition, considerable improvements might be achieved by implementing and sustaining the strategies of the ‘baby friendly community initiatives’ given the high proportion of home births in Nigeria, including broader national policies that support mothers in work environments. Facility based programs that underpin family planning, focused antenatal care and quality early essential newborn care are proposed to promote breastfeeding practices among women, acknowledging that a health service contact presents an important opportunity for the implementation of breastfeeding promotion and support. Finally, intervention studies that evaluate current policy initiatives addressing maternal and child health outcomes, and the wider impacts of health system strengthening should be key priorities in improving infant feeding patterns among Nigerian women.

## Additional file

Additional file 1: Table S1. Prevalences of exclusive breast feeding, predominant breastfeeding, bottle-feeding and early initiation of breastfeeding by socio-economic, child and health service characteristics, Nigeria 2008.

## References

[1] Ladomenou F, Moschandreas J, Kafatos A, Tselentis Y, Galanakis E (2010). Protective effect of exclusive breastfeeding against infections during infancy: a prospective study. Arch Dis Child.

[2] World Health Organization (2000). WHO Collaborative study team on the role of breastfeeding on the prevention of infant mortality effect of breastfeeding on infant and child mortality due to infectious diseases in less developed countries: a pooled analysis. Lancet.

[3] World Health Organisation (2003). The global strategy for infant and young child feeding.

[4] Ip S, Chung M, Raman G, Chew P, Magula N, DeVine D (2007). Breastfeeding and maternal and infant health outcomes in developed countries.

[5] Dezoysa RM, Martines J (1991). Why promote feeding in diarhhoea disease control programmes. Health Policy Plan.

[6] World Health Organization. Indicators for accessing breastfeeding practices. In: WHO/CDD/SER/91.1, editor. 1991, Geneva: World Health Organization.

[7] World Health Organization. Infant and young child nutrition: global strategy for infant and young child feeding. 2001 [cited 2014 19/01/2014]; Available from: [http://apps.who.int/gb/archive/pdf_files/WHA55/ea5515.pdf]

[8] World Health Organization. The state of breastfeeding in 33 countries. 2010 22/01/2014]; Available from: http://www.worldbreastfeedingtrends.org.

[9] Kramer MS, Kakuma R (2004). The optimal duration of exclusive breastfeeding: a systematic review. Adv Exp Med Biol.

[10] United Nations Children’s Fund (UNICEF) (2013). BREASTFEEDING ON THE WORLDWIDE AGENDA Findings from a landscape analysis on political commitment for programmes to protect, promote and support breastfeeding.

[11] Black RE, Victora CG, Walker SP, Bhutta ZA, Christian P, De Onis M (2013). Maternal and child undernutrition and overweight in low-income and middle-income countries. Lancet.

[12] Black R, Morris SS, Bryce J, The Maternal and Child Undernutrition Study Group (2008). Maternal and child undernutrition: global and regional exposures and health consequences. Lancet.

[13] Ogunlesi T, Dedeke O, Okeniyi J, Oyedeji G (2004). Infant and toddler feeding practices in the Baby Friendly Initiative (BFI) Era In Ilesa, Nigeria. Int J Nutr Wellness.

[14] National Population Commission (NPC) [Nigeria] and ICF Macro (2013). Nigeria Demographic and Health Survey 2013.

[15] Ogunlesi TA (2010). Maternal socio-demographic factors influencing the initiation and exclusivity of breastfeeding in a Nigerian semi-urban setting. Matern Child Health J.

[16] Agho K, Dibley E, Odiase MJ, Ogbonmwan JI, Sunday M (2011). Determinants of exclusive breastfeeding in Nigeria. BMC Pregnancy Childbirth.

[17] Yahya BW, Adebayo BS (2003). Modelling the trend and determinants of breastfeeding initiation in Nigeria. Child Dev Res.

[18] Center for Disease Control and Prevention (2012). Breastfeeding Report Card 2012, United States: outcome indicators.

[19] Victor R, Baines SK, Agho KE, Dibley MJ (2013). Determinants of breastfeeding indicators among children less than 24 months of age in Tanzania: a secondary analysis of the 2010 Tanzania Demographic and Health Survey. BMJ Open.

[20] Kenya National Bureau of Statistics, MEASURE DHS, and ICF Macro (2010). Kenya Demographic and Health Survey, 2008–09.

[21] World Health Organization (2010). Data bank on infant and young child feeding on Nigeria.

[22] Salami L (2006). Factors influencing breastfeeding practices in Edo state, Nigeria. Afr J Food Agric Nutr Dev.

[23] Ekure EN, Antia-Obong ON, Udo JJ, Edet EE (2003). Maternal exclusive breastfeeding practice in Calabar Nigeira: some related social characteristics. Nigeria J Clin Pract.

[24] Maduforo AN, Ubah NC, Obiakor–okeke PN (2013). The practice of exclusive breastfeeding by lactating women in Owerri metropolis, Imo State, Nigeria. Global Adv Res J Med Med Sci.

[25] Ukegbu AU, Ukegbu PO, Onyeonoro UU, Ubajaka CF (2011). Determinants of breastfeeding patterns among mothers in Anambra State, Nigeria. South Afr J Child Health.

[26] Qureshi AM, Oche OM, Sadiq UA, Kabiru S (2011). Using community volunteers to promote exclusive breastfeeding in Sokoto State, Nigeria. Pan Afr Med J.

[27] Agunbiade OM, Ogunleye OV (2012). Constraints to exclusive breastfeeding practice among breastfeeding mothers in Southwest Nigeria: implications for scaling up. Int Breastfeed J.

[28] Sadoh AE, Sadoh WE, Oniyelu P (2011). Breast feeding practice among medical women in Nigeria. Nigeria Med J.

[29] National Population Commission (NPC) [Nigeria] and ICF Macro (2008). Nigeria Demographic and Health Survey 2008.

[30] World Health Organisation (2008). Indicators for assessing infant and young child feeding practices.

[31] WHO, U., USAID, FANTA, AED, UC DAVIS, IFPRI (2010). Indictors for assessing infant and young child feeding practices part III.

[32] National Population Commission (NPC) [Nigeria] and ICF Macro (1999). Nigeria Demographic and Health Survey 1999.

[33] National Population Commission (NPC) [Nigeria] and ICF Macro (2003). Nigeria Demographic and Health Survey 2003.

[34] Filmer D, Pritchett LH (2001). Estimating wealth effects without expenditure data—or tears: an application to educational enrollments in states of India. Demography.

[35] World Health Organization (1978). Primary Health Care, Report of the International conference on primary health care, Alma-Ata, USSR, 6–12 September, 1978.

[36] Khanal V, Sauer K, Zhao Y (2013). Determinants of complementary feeding practices among Nepalese children aged 6–23 months: findings from demographic and health survey 2011. BMC Pediatr.

[37] Akram HT, Dure-Samin N, Yasir B, Kazmi N, Agho KE, Abbasi S (2013). Determinants of suboptimal breast-feeding practices in Pakistan. Public Health Nutr.

[38] Tawiah-Agyemang C, Kirkwood BR, Edmond K, Bazzano A, Hill Z (2008). Early initiation of breast-feeding in Ghana: barriers and facilitators. J Perinatol.

[39] Edmond KM, Zandoh C, Quigley MA, Amenga-Etego S, Owusu-Agyei S, Kirkwood BR (2006). Delayed breastfeeding initiation increases risk of neonatal mortality. Pediatrics.

[40] Dubois L, Girard M (2003). Social determinants of initiation, duration and exclusivity of breastfeeding at the population level: the results of the longitudinal study of child development in Quebec (ELDEQ 1998–2002). Can J Public Health.

[41] Kaunonen M, Hannula L, Tarkka MT (2012). A systematic review of peer support interventions for breastfeeding. J Clin Nurs.

[42] Babalola S, Fatusi A (2009). Determinants of use of maternal health services in Nigeria-looking beyond individual and household factors. BMC Pregnancy Childbirth.

[43] Simkhada B, Teijlingen ER, Porter M, Simkhada P (2008). Factors affecting the utilization of antenatal care in developing countries: systematic review of the literature. J Adv Nurs.

[44] Gertler P, Rahman O, Feifer C, Ashley D (1993). Determinants of pregnancy outcomes and targeting of maternal health services in Jamaica. Soc Sci Med.

[45] Centers for Disease Control and Prevention (CDC). HIV/AIDS among US women: Minority and young women at continuing risk. Atlanta, GA: Centers for Disease Control and Prevention (2002).

[46] Ugboaja JO, Berthrand NO, Igwegbe OA, OBI-Nwosu AL (2013). Barriers to postnatal care and exclusive breastfeeding among urbanwomen in southeastern Nigeria. Nigerian Med J.

[47] Alutu ANG, Orubu OA (2005). Barriers to successful exclusive breast-feeding practices among rural and urban nursing mothers in Edo State of Nigeria: Implications for education and counselling. Res Rev.

[48] Agbo HA, Envuladu EA, Adams HS, Inalegwu E, Okoh E, Agba A (2013). Barriers and facilitators to the practice of exclusive breast feeding among working class mothers: a study of female resident doctors in tertiary health institutions in Plateau State. J Med Res.

[49] Wole O (2013). Why breastfeeding is becoming unpopular, by mothers in The Guardian Nigeria.

[50] Aidam BA, Perez-Escamilla R, Lartey A, Aidam J (2005). Factors associated with exclusive breastfeeding in Accra, Ghana. Eur J Clin Nutr.

[51] World Health Organization, UNICEF, and University Of California. Complementary feeding of young children in developing countries: a review of current scientific knowledge. In: Brown K, Dewey K and Allen L, editors. 1998. Geneva: World Health Organization.

[52] Bezner KR, Dakishoni L, Shumba L, Msachi R, Chirwa M (2008). “We grandmothers know plenty”: breastfeeding, complementary feeding and the multifaceted role of grandmothers in Malawi. Soc Sci Med.

[53] Grassley J, Eschiti V (2008). Grandmother breastfeeding support: what do mothers need and want?. Birth.

[54] Olufowobi S (2014). Lagos okays six-month maternity leave, 10 days for fathers, in The Punch.

[55] Aghaji M (2001). Exclusive breast-feeding practice and associated factors in Enugu, Nigeria. West Afr J Med.

[56] Maycock B, Binns CW, Dhaliwal S, Tohotoa J, Hauck Y, Burns S (2013). Education and support for fathers improves breastfeeding rates a randomized controlled trial. J Hum Lact.

[57] Dearden K, Mekibib A, de Irma M, de Maritza O, Stone-Jimenez M, Morrow AL (2002). Determinants of optimal breast-feeding in peri-urban Guatemala City, Guatemala. Rev Panam Salud Publica.

[58] Prior E, Santhakumaran S, Gale C, Philipps LH, Modi N, Hyde MJ (2012). Breastfeeding after cesarean delivery: a systematic review and meta-analysis of world literature. Am J Clin Nutr.

[59] World Health Organisation, United Nation Childen Education Fund (2009). Baby-friendly hospital initiative revised, updated and expanded for integrated care.

[60] Akmam W (2002). Women’s education and fertility rates in developing countries, with special reference to Bangladesh. Eubios J Asian Int Bioethics.

[61] ACS Distance Education. How can society influences health. n.d [cited 2014 11th December,]; Available from: http://www.acs.edu.au/info/natural-health/mental/social-influences.aspx (Accessed December, 2014).

[62] Van Der Meulen Rodgers Y (2011). Maternal employment andchild health.

[63] Patel A, Badhoniya N, Khadse S, Senarath U, Agho KE, Dibley M (2010). Infant and young child feeding indicators and determinants of poor feeding practices in India: secondary data analysis of National Family Health Survey 2005–06. Food Nutri Bull.

[64] Federal Government of Nigeria. Mellinium Development Goals Performance Tracking Survey 2012. In: National Bureau of Statistics, editor. 2013. Abuja, Nigeria: Federal Government of Nigeria.

[65] Campbell, J., Why Nigeria’s North South Distinction Is Important, in The World Post. 2011, The Huffington Post and The Berggruen Institute of Governance: Online; available at http://www.huffingtonpost.com/amb-john-campbell/why-nigerias-north-south-_b_817734.html (Accessed November, 2014).

