# Supplementary material for: Determinants of suboptimal breastfeeding practices in Nigeria: evidence from the 2008 demographic and health survey
Source: BMC Public Health. 2015 Mar 18;15:259. doi: 10.1186/s12889-015-1595-7 (PMC4367831; doi:10.1186/s12889-015-1595-7)
Supplement: Additional file 1: Table S1. — Prevalences of exclusive breast feeding, predominant breastfeeding, bottle-feeding and early initiation of breastfeeding by socio-economic, child and health service characteristics, Nigeria 2008. [file 12889_2015_1595_MOESM1_ESM.doc]

**Supplementary table: Prevalences of exclusive breast feeding, predominant breastfeeding, bottle-feeding and e**arly initiation of breastfeeding by socio-economic, child and health service characteristics, Nigeria 2008

|  |  | **Exclusive breastfeeding** |  | **Predominant breastfeeding** |  | **Bottle-feeding** |  | **Early initiation of breastfeeding** |  |
| --- | --- | --- | --- | --- | --- | --- | --- | --- | --- |
|  | **N** | **%[95% CI]** | **p** | **%[95% CI]** | **p** | **%[95% CI]** | **p** | **% [95% CI]** | **p** |
| ***Socio-economic*** |  |  |  |  |  |  |  |  |  |
| **Work in the last 12 months** |  |  |  |  |  |  |  |  |  |
| Not working | 3978 | 11.9[9.8,14.4] | 0.156 | 50.5[47.1,53.9] | 0.126 | 9.9[8.8,11.2] | 0.014 | 34.1[31.7,36.5] | <0.001 |
| Working | 6220 | 14.6[12.6,16.8] |  | 46.1[42.9,49.2] |  | 12[11.0,13.1] |  | 40.0[38.0,42.1] |  |
| **Maternal education** |  |  |  |  |  |  |  |  |  |
| No education | 4655 | 7.4[5.6,9.7] | <0.001 | 55.9[52.2,59.6] | <0.001 | 4.4[3.8,5.2] | <0.001 | 30.4[28.1,32.8] | <0.001 |
| Primary | 2274 | 14.0[11.2,17.3] |  | 45.1[40.7,49.6] |  | 13.4[11.8,15.2] |  | 43.7[40.7,46.7] |  |
| Secondary and above | 3296 | 21.0[18.1,24.2] |  | 39.8[36.2,43.6] |  | 19.2[17.6,21.0] |  | 43.9[41.5,46.3] |  |
| **Father's education** |  |  |  |  |  |  |  |  |  |
| No education | 3641 | 6.8[4.9,9.5] | <0.001 | 55.6[51.8,59.4] | <0.001 | 3.7[3.1,4.5] | <0.001 | 30.3[27.7,32.9] | <0.001 |
| Primary | 2149 | 11.5[8.8,14.7] |  | 49.0[44.0,54.0] |  | 12.5[10.8,14.4] |  | 39.1[36.2,42.2] |  |
| Secondary | 4044 | 19.7[17.1,22.6] |  | 42.2[38.8,45.6] |  | 17[15.6,18.5] |  | 43.8[41.6,46.0] |  |
| **Mother's age** |  |  |  |  |  |  |  |  |  |
| 15-24 years | 3112 | 10.3[8.5,12.6] | <0.001 | 50.6[46.7,54.5] | 0.230 | 11.4[10.1,12.9] | 0.111 | 33.8[31.6,36.1] | <0.001 |
| 25-34 years | 5019 | 16.3[13.9,19.0] |  | 46.9[43.8,50.0] |  | 11.6[10.5,12.8] |  | 39.8[37.8,41.8] |  |
| 35-49 years | 2093 | 11.7[8.9,15.3] |  | 46.6[41.9,51.5] |  | 9.7[8.3,11.4] |  | 38.4[35.7,41.1] |  |
| **Household wealth** |  |  |  |  |  |  |  |  |  |
| Poorest | 4709 | 7.7[6.0,9.8] | <0.001 | 54.0[50.3,57.6] | <0.001 | 5.9[5.1,6.9] | <0.001 | 32.8[30.3,35.4] | <0.001 |
| Middle | 3749 | 16.6[14.0,19.6] |  | 43.8[40.5,47.2] |  | 13.3[12.1,14.7] |  | 41.8[39.5,44.1] |  |
| Richest | 1767 | 21.9[17.8,26.7] |  | 41.6[36.1,47.4] |  | 20.5[18.3,23.0] |  | 42.1[38.8,45.5] |  |
| **Geopolitical region** |  |  |  |  |  |  |  |  |  |
| North Central | 3112 | 21.6[17.2,26.6] | <0.001 | 30.6[26.4,35.2] | <0.001 | 11.1[9.0,13.6] | <0.001 | 59.6[55.0,63.9] | <0.001 |
| North East | 5019 | 4.0[2.7,5.8] |  | 60.5[54.3,66.3] |  | 6.8[5.5,8.4] |  | 23.8[20.2,27.8] |  |
| North West | 2093 | 8.4[5.6,12.4] |  | 62.8[58.0,67.3] |  | 4.1[3.2,5.2] |  | 30.6[27.6,33.7] |  |
| South East | 3112 | 14.9[11.1,19.7] |  | 42.3[36.1,48.8] |  | 20.6[17.6,23.9] |  | 38.3[34.2,42.4] |  |
| South West | 5019 | 16.5[12.5,21.4] |  | 28.2[23.1,33.9] |  | 19.5[16.8,22.6] |  | 50.2[45.1,55.3] |  |
| South South | 2093 | 22.3[17.6,27.7] |  | 43.5[37.6,49.5] |  | 16.5[14.1,19.2] |  | 36.7[32.9,40.5] |  |
| ***Individual*** |  |  |  |  |  |  |  |  |  |
| **Age of child (months)** |  |  |  |  |  |  |  |  |  |
| 0-5 | 2832 | 13.4[11.9,15.2] | <0.001 | 48.1[45.7,50.5] | <0.001 | 15.3[13.7,17.1] | <0.001 | 37.1[34.9,39.4] | 0.817 |
| 6-11 | 2772 |  |  |  |  | 13.4[12.0,15.0] |  | 38.4[36.0,40.9] |  |
| 12-17 | 2806 |  |  |  |  | 8.4[7.2,9.6] |  | 37.4[35.0,39.8] |  |
| 18-23 | 1815 |  |  |  |  | 5.7[4.7,7.0] |  | 38.0[35.1,41.0] |  |
| **Birth order** |  |  |  |  |  |  |  |  |  |
| First-born | 1953 | 14.6[11.6,18.2] | 0.061 | 46.6[41.9,51.3] | 0.151 | 15.8[13.9,17.9] | 0.001 | 34.4[31.9,37.1] | 0.010 |
| 2nd-4^th^ | 4703 | 14.7[12.6,16.9] |  | 46.6[43.5,49.8] |  | 11.9[10.8,13.1] |  | 38.7[36.8,40.7] |  |
| 5 or more | 3569 | 11.3[9.1,13.8] |  | 50.8[47.1,54.5] |  | 7.7[6.7,8.8] |  | 38.1[35.8,40.5] |  |
| **Preceding birth interval** |  |  |  |  |  |  |  |  |  |
| No previous birth | 1953 | 14.6[11.6,18.2] | 0.515 | 46.6[41.9,51.3] | 0.319 | 15.8[13.9,17.9] | <0.001 | 34.4[31.9,37.1] | 0.007 |
| <24 months | 1591 | 11.6[8.4,15.9] |  | 46.5[41.0,52.1] |  | 11.7[9.9,13.9] |  | 36.5[33.7,39.5] |  |
| ≥24 months | 6668 | 13.4[11.7,15.3] |  | 48.9[46.2,51.6] |  | 9.7[8.8,10.6] |  | 38.9[37.0,40.9] |  |
| ***Health service*** |  |  |  |  |  |  |  |  |  |
| **Combined place and mode of delivery** |  |  |  |  |  |  |  |  |  |
| Home | 6563 | 42.9[6.3,89.4] | 0.008 | 55.0[46.7,62.2] | 0.342 | 13.1[1.8,55.8] | <0.001 | 37.7[36.0,39.4] | 0.005 |
| Health facility with non-caesarean | 3481 | 13.2[11.6,14.9] |  | 48.2[45.8,50.6] |  | 10.9[10.0,11.8] |  | 37.9[36.2,39.6] |  |
| Health facility with caesarean | 175 | 27.0[16.2,41.5] |  | 45.1[30.0,61.1] |  | 28.8[22.0,36.8] |  | 25.5[18.4,34.3] |  |
| **Type of delivery assistance** |  |  |  |  |  |  |  |  |  |
| Health professional | 3390 | 19.6[16.7,22.7] | <0.001 | 42.6[38.9,46.3] | 0.004 | 18.3[16.7,20.0] | <0.001 | 43.9[41.5,46.3] | <0.001 |
| Traditional birth assistants | 2208 | 7.1[4.8,10.3] |  | 50.9[45.7,56.1] |  | 8.0[6.7,9.6] |  | 36.0[32.8,39.5] |  |
| Other unskilled personnel | 2678 | 12.9[10.4,15.9] |  | 47.8[43.9,51.7] |  | 10[8.7,11.5] |  | 36.5[33.8,39.2] |  |
| No one | 1949 | 9.8[6.5,14.7] |  | 55.5[50.0,60.8] |  | 4.1[3.1,5.3] |  | 30.4[27.0,34.1] |  |
| **Antenatal Clinic visits** |  |  |  |  |  |  |  |  |  |
| None | 4594 | 9.1[7.2,11.5] | <0.001 | 54.7[51.1,58.3] | <0.001 | 6.0[5.2,6.9] | <0.001 | 35.1[32.7,37.5] | 0.002 |
| 1-3 | 1084 | 11.2[8.0,15.6] |  | 43.1[37.5,48.9] |  | 11.9[9.8,14.3] |  | 39.6[35.7,43.6] |  |
| ≥4 | 4547 | 18.4[15.9,21.2] |  | 42.8[39.4,46.2] |  | 16.3[14.9,17.7] |  | 39.9[37.8,42.0] |  |
| **Timing of postnatal (PNC) check-up** |  |  |  |  |  |  |  |  |  |
| No PNC | 5914 | 9.9[8.1,12.0] | <0.001 | 52.6[49.5,55.6] | <0.001 | 6.9[6.1,7.8] | <0.001 | 34.6[32.4,36.8] | <0.001 |
| 0-2 days | 2600 | 17.3[14.4,20.7] |  | 44.6[40.2,49.1] |  | 15.3[13.6,17.2] |  | 44.8[42.0,47.6] |  |
| 3-6 days | 628 | 23.2[17.0,30.9] |  | 37.6[30.2,45.8] |  | 20.4[16.8,24.4] |  | 44.2[39.5,49.1] |  |
| 7+ days | 1083 | 16.7[12.1,22.5] |  | 39.0[32.8,45.7] |  | 19.1[16.4,22.2] |  | 34.0[30.4,37.7] |  |
